# Case of Twins, in Which Premature Labour Was Induced by Redundancy of Liquor Amnii

**Published:** 1818-07-01

**Authors:** J. B. Sheppard

**Affiliations:** Member of the Royal College of Surgeons in London, and Surgeon in the Royal Navy


*Case of Twins, in which premature Labour was induced by redundancy of Liquor Amnii.* By J. B. SHEPPARD, Member of the Royal College of Surgeons in London, and Surgeon in the Royal Navy.

Mrs. H. aged 30, the mother of three children, consulted me on the 3d of December last, on account of a distressing degree of dyspnœa, with pain in the left side, and frequent cough. She distinctly stated that she was in the last month of her pregnancy, and her appearance certainly afforded no reason to doubt the correctness of the allegation. The pulse indicating considerable febrile irritation, a moderate quantity of blood was taken from the arm, and a laxative given. As she resided in a distant village, I did not see her again until the 16th, when she had attained a most unweildly size; the consequent progressive aggravation of her symptoms rendering her wholly incapable of supporting the recumbent position. It being evident that her sufferings depended solely upon the diminution of the capacity of the thorax, from the pressure of the enormous uterine tumour, it was improbable that she would derive relief from medicine; she was therefore enjoined perfect rest, and her attention directed to her approaching delivery, as the period of her release from her afflictions.

On the evening of the 18th, labour pains supervened. On my arrival at ten o'clock, I found the os uteri partially dilated; but the presenting part of the child could not be felt, as the protruding membranes neither receded, nor became flaccid during the absence of pain, but continued to present a tense, incompressible, conical tumour. At four in the morning, the os uteri was pretty fully dilated, and the apex of the membranous tumour reached the os externum. I then considered it proper to rupture the membranes, as from their sensible density of texture, there was no reason to expect their laceration by the effect of the uterine contraction alone; and it became important to ascertain the presentation. A deluge of liquor amnii succeeded their rupture with the finger; the distended abdomen instantly sunk to comparative flatness, and the patient expressed herself perfectly relieved from the dyspnœa with which she had so long contended. A slight degree of hæmorrhage supervened towards the conclusion of the discharge of the liquor amnii. On examination, the feet of the child were found to present; and by the effect of a few pains a living child was delivered: but it was obvious that the statement of the completion of the full period of gestation was erroneous; the child not exceeding the growth of one of seven months. The funis was scarcely divided, before a placenta was expelled by a pain; but it was ascertained to be unconnected with the child already delivered; and accordingly, on examination, the breech of a second child was found to present. The hæmorrhage was become considerable, and the case assumed a more complex and serious character. The principal indication was to bring the uterus into a condition to allow of complete contraction; the hand, therefore, was passed into the uterus, and the feet brought slowly down: the delivery was accomplished without difficulty, and the child (which was also a female, and born alive, though extremely feeble), was separated from the placenta previously expelled. From the retention of the placenta of the first child, and consequent incomplete contraction of the uterus, the hæmorrhage continued; no time, therefore, was lost in again introducing the hand into the uterus, the stimulus of which produced contraction of that organ, and detached the remaining placenta. The hand was retained a short time in the uterus, with the view of exciting forcible and effectual contraction, and the placenta was then slowly withdrawn: the hæmorrhage shortly ceased, and nothing unfavourable subsequently occurred to interrupt the progress to perfect recovery. The first child survived delivery about half an hour; the second not more than ten minutes.

There can be no doubt that in the above case the premature developement of the uterus by the morbid redundance of the liquor amnii, was the immediate cause of the supervention of premature labour. The inaccuracy of the reckoning may be explained by the admission of the patient after delivery, that she had permitted herself to be misled by her extraordinary size, which she could not refer to any other cause than that of having attained the full period of gestation.

Witney, Oxfordshire, March 12, 1818.

